# Group-Delivered Mindfulness-Based Cognitive Therapy to Reduce Psychological Distress and Improve Sleep in Patients With Inflammatory Bowel Diseases: A Multicenter Randomized Controlled Trial (MindIBD)

**DOI:** 10.1093/ibd/izaf116

**Published:** 2025-07-14

**Authors:** Milou M ter Avest, Marloes J Huijbers, Carmen S Horjus, Tessa E H Römkens, Ellen M Witteman, Willemijn A van Dop, Martin Dresler, A Rogier T Donders, Gerard Dijkstra, Loes H C Nissen, Anne E M Speckens

**Affiliations:** Department of Psychiatry, Centre for Mindfulness, Radboud University Medical Centre, Nijmegen, the Netherlands; Donders Institute for Brain, Cognition and Behaviour, Radboud University Medical Centre, Nijmegen, the Netherlands; Department of Gastroenterology and Hepatology, Jeroen Bosch Hospital, ‘s-Hertogenbosch, the Netherlands; Department of Psychiatry, Centre for Mindfulness, Radboud University Medical Centre, Nijmegen, the Netherlands; Department of Gastroenterology and Hepatology, Rijnstate Hospital, Arnhem, the Netherlands; Department of Gastroenterology and Hepatology, Jeroen Bosch Hospital, ‘s-Hertogenbosch, the Netherlands; Department of Gastroenterology and Hepatology, Canisius Wilhelmina Hospital, Nijmegen, the Netherlands; Department of Gastroenterology and Hepatology, Radboud University Medical Centre, Nijmegen, the Netherlands; Donders Institute for Brain, Cognition and Behaviour, Radboud University Medical Centre, Nijmegen, the Netherlands; Department IQ Health, Radboud University Medical Centre, Radboud Institute for Health Sciences, Nijmegen, the Netherlands; Department of Gastroenterology and Hepatology, University Medical Centre Groningen, University of Groningen, Groningen, the Netherlands; Department of Gastroenterology and Hepatology, Jeroen Bosch Hospital, ‘s-Hertogenbosch, the Netherlands; Department of Psychiatry, Centre for Mindfulness, Radboud University Medical Centre, Nijmegen, the Netherlands; Donders Institute for Brain, Cognition and Behaviour, Radboud University Medical Centre, Nijmegen, the Netherlands

**Keywords:** randomized controlled trial, Mindfulness-Based Cognitive Therapy, inflammatory bowel diseases, psychological distress

## Abstract

**Background:**

Many patients with inflammatory bowel disease (IBD) suffer from psychological distress, sleep disturbances, fatigue, and a reduced quality of life—even during remission. Mindfulness-Based Cognitive Therapy (MBCT) has been effective in other populations, and therefore it may also benefit IBD patients. The primary objective was to evaluate the effectiveness of MBCT plus treatment as usual (TAU) in reducing psychological distress compared to TAU alone.

**Methods:**

This multicenter randomized controlled trial study included IBD patients in remission, aged 16 and older, who experienced at least mild levels of psychological distress (Hospital Anxiety and Depression Scale ≥ 11). Assessments were conducted at baseline, post-intervention (3 months), and at 6, 9, and 12 months after baseline. This trial was registered at ClinicalTrials.gov: NCT04646785.

**Results:**

A total of 142 participants were allocated to MBCT + TAU (*n* = 70) or TAU alone (*n* = 72). Group-delivered MBCT significantly reduced psychological distress (*d* = −0.61) and improved well-being (*d* = 0.40) post-intervention, with consolidation of the effects over time. Exploratory objective sleep metrics (ie, electroencephalography) showed a reduction in total sleep time (*d* = 0.67) after MBCT with an increase in the proportion of deep sleep (*d* = 0.70). While flare occurrence showed no difference, fecal calprotectin levels reduced in the MBCT + TAU group over the follow-up period (*d* = −0.49).

**Conclusions:**

Mindfulness-Based Cognitive Therapy can be considered a valuable addition to the limited effective psychosocial interventions set for IBD patients, as it reduces psychological distress and improves well-being. This study also shows the possible impact of MBCT on biological processes, such as sleep and inflammation.

**Key Messages**

**What is already known?**

Inflammatory bowel diseases (IBD) are associated with psychological distress, fatigue, sleep disturbances, and reduced quality of life. Growing evidence supports the bidirectional links between psychological distress, poor sleep, and disease activity.

**What is new here?**

This study shows that Mindfulness-Based Cognitive Therapy (MBCT) is effective in reducing psychological distress, with consolidation of the effect at long term. Additionally, it also demonstrates the possible impact of MBCT on biological processes, such as sleep and inflammation.

**How can this study help patient care?**

Based on these findings, MBCT should be added as a psychological treatment option for IBD patients experiencing psychological distress.

## Introduction

Inflammatory bowel disease (IBD), mainly consisting of Crohn’s disease (CD) and ulcerative colitis (UC), is characterized by its unpredictable relapsing-remitting course and is typically treated with long-term immunosuppressive medication and surgery. In addition to associated physical symptoms, such as abdominal pain and bloody diarrhea, the disease is frequently accompanied by psychological distress (eg, symptoms of anxiety and depression), fatigue, and sleep disturbances.^1–3^ Moreover, these factors contribute to a reduced quality of life (QoL), poorer self-care, and increased utilization of medical care.^4,5^ Although disease activity aggravates these issues, mental health problems persist even during periods of inactive disease, and may, in turn, also affect the disease course.^6^ Additionally, the evidence for a bidirectional relationship between poor sleep quality and disease activity is growing.^7^ This interconnection between both mental health and sleep quality with disease progression emphasizes the need for interventions that address these issues.

Mindfulness-based interventions (MBIs) have been shown to reduce psychological distress and fatigue while improving sleep quality and QoL in patients with both psychiatric and somatic conditions.^8^ During MBIs, individuals learn to be more aware of present moment experiences without judgment, thereby fostering recognition of unhelpful patterns in thoughts and behavior.^9^ The effect of MBIs on psychological distress is potentially mediated by mechanisms such as repetitive negative thinking, mindfulness skills, self-compassion.^10^ Additionally, sleep could be a contributing factor, as poor sleep is linked to higher psychological distress,^11^ and MBIs have the potential to enhance sleep quality.^12^ Examples of evidence-based MBIs are the 8-week group-based programs Mindfulness-Based Stress Reduction (MBSR) and Mindfulness-Based Cognitive Therapy (MBCT).

Interest in MBIs is growing within IBD care, and its effectiveness on perceived stress and QoL is promising.^13,14^ Nevertheless, the effectiveness of MBIs on psychological distress in IBD varies according to recent meta-analyses.^13,14^ Additionally, only two of the included studies assessed subjective sleep quality as an outcome measure, both reporting no improvement.^15,16^ There are no studies available in which sleep was measured objectively in IBD patients. Two studies interested in fatigue as an outcome measure showed positive short-term results.^17,18^ From the perspective that stress and psychological distress can negatively impact disease progression, MBCT could hypothetically have a positive effect on disease activity. However, studies that included flares or fecal calprotectin as outcome measures have shown inconsistent results.^13,14^

The primary aim of the MindIBD trial is to strengthen the evidence for the effectiveness of MBCT in reducing psychological distress post-intervention in patients with IBD. Secondary outcome measures include well-being, sleep, fatigue, and disease activity. Sleep and disease activity are assessed with both subjective and objective measures. Treatment outcomes are assessed both post-intervention and over a 1-year follow-up period. In addition, moderation analyses are performed to examine whether specific subgroups benefited more from MBCT than others. Finally, we investigate whether repetitive negative thinking, mindfulness skills, self-compassion, and sleep quality mediate the effect of MBCT on psychological distress.

## Materials and Methods

### Study Design

The MindIBD trial is a multicenter randomized controlled trial (RCT) comparing MBCT plus treatment as usual (TAU) with TAU alone using a follow-up period of 12 months. Participating hospitals included one university hospital (Radboud University Medical Centre in Nijmegen) and three general hospitals (Jeroen Bosch Hospital in ‘s-Hertogenbosch, Canisius Wilhelmina Hospital in Nijmegen, and Rijnstate Hospital in Arnhem). The study was approved by the medical ethical review board Eastern Netherlands (#2021-7319; NL75762.091.20), and its protocol has been previously published.^19^

### Participants

Patients were eligible for participation when they met the following inclusion criteria: (1) minimum age of 16 years; (2) physician-confirmed IBD diagnosis; (3) IBD in remission for at least 3 months, defined by fecal calprotectin < 250 mg/kg before allocation,^20^ and no IBD medication or being on a stable dose for at least 3 months; (4) Hospital Anxiety and Depression Scale (HADS) total score ≥ 11; and (5) good understanding of the Dutch language. Exclusion criteria were: (1) severe psychiatric disorders; (2) substance use disorders; (3) anemia; (4) prior participation in an 8-week MBI. In addition, we decided to exclude pregnant women or women actively planning pregnancy due to its potential impact on fatigue and well-being.

### Procedure and Assessments

Between July 2021 and May 2022, all IBD patients from the participating hospitals received an invitation letter with information about the trial. Interested patients were referred to an online anonymous prescreening survey. Eligible patients were directed to an online registration form, after which they received additional study information and were invited for a research interview. During this interview, in- and exclusion criteria were reviewed, and written informed consent was obtained. Laboratory tests were conducted to rule out anemia or elevated fecal calprotectin levels during 8 weeks before the start of the study period.

Subsequently, eligible participants were invited for the baseline assessment (T0), which included demographic- and disease-specific data collection, self-report questionnaires, and an objective sleep assessment. The objective sleep assessment was conducted using a headband (Zmax, Hypnodyne) equipped with gel pad electrodes that were applied to the forehead to record 2-channel electroencephalography (EEG). Participants were instructed on its use during the research interview, were provided with written information, and had easy access to support from the research team in case of any practical issues. Participants were asked to record their sleep for three consecutive nights at home. The post-intervention assessment (3 months after baseline, T1) and the follow-up assessments at 6, 9, and 12 months after baseline (T2, T3, and T4, respectively) consisted of self-report questionnaires and a clinician-administered interview about current disease activity (Harvey-Bradshaw Index [HBI] and Simple Clinical Colitis Activity Index [SCCAI]), IBD course (eg, flares and medication changes), and adverse events. The objective sleep assessment was repeated for another three consecutive nights at T1. At T2 and T4, blood and stool samples were included.

### Randomization and Blinding

Participants were randomly assigned in a 1:1 ratio to either MBCT + TAU or TAU alone after the baseline assessment was completed. Randomization was stratified by hospital, sex, and IBD type, using block randomization with varying block sizes (2, 4, 6), and was electronically performed via CastorEDC. The coordinating researcher (MtA) was blinded for block sizes. The organization of the trial did not allow for further blinding of the coordinating researcher, who performed recruitment, communication of the allocated arms, and data collection. By nature of the intervention, patients were also not blinded to the condition they had been randomized.

### Intervention

#### Mindfulness-based cognitive therapy

The MBCT program was based on the original protocol developed by Segal et al.,^21^ with slight adjustments for IBD patients (Table S1). The program consisted of eight weekly group-delivered sessions of 2.5 hours, with an additional 30-45 minutes of daily home practice and a silent day. The silent day was a 6-hour retreat-like session, taking place between sessions 6 and 7, during which a variety of guided meditation exercises were combined with mindful walking and eating in silence. A total of nine MBCT groups started between September 2021 and October 2022, with group sizes ranging from 8 to 12 participants. Despite the COVID-19 pandemic, all groups were taught in person, with an option to join online if participants had tested positive or had symptoms. Each group was led by a certified mindfulness teacher, in accordance with the advanced criteria of the Association of Mindfulness-Based Teachers in the Netherlands and Flanders.^22^ The four teachers were a psychiatrist, psychologist, spiritual counselor, and psychomotor therapist. All mindfulness teachers attended a kick-off meeting before the trial, where the trial-specific MBCT protocol was discussed. In addition, peer supervision meetings were organized twice per MBCT course. Sessions 2 to 8 were video recorded for assessment of protocol adherence and teacher competence. For each of the four teachers, two sessions were randomly selected and independently rated by two experienced raters using the Mindfulness-Based Interventions: Teaching Assessment Criteria (MBI:TAC).^23^ One trainer was rated as an advanced beginner, one as competent, and two as proficient.

#### Treatment as usual

Treatment as usual is mainly focused on pharmacological and surgical disease control treatments and prevention of complications, according to Dutch and European treatment guidelines.^24–27^ Participants in the TAU alone group were requested not to start MBIs during the study period. Other psychological treatments (already ongoing or newly started) were not prohibited. All healthcare use was reported during the clinician-administered interviews. Participants in the TAU alone group were able to participate in MBCT after completion of the study.

### Outcomes

The outcome measures are discussed in more detail in the study protocol paper.^19^ The primary outcome was psychological distress at post-intervention, measured by the HADS total score.

Other mental health outcomes were repetitive negative thinking (Perseverative Thinking Questionnaire), mindfulness skills (Five Facet Mindfulness Questionnaire-Short Form), self-compassion (Self-Compassion Scale-Short Form), and well-being (Mental Health Continuum-Short Form, MHC-SF).

The concept of sleep quality encompasses both objective and subjective dimensions. Objectively, it can be assessed through sleep duration, sleep continuity, and sleep architecture. Subjectively, it includes self-perceived satisfaction with sleep and its impact on daily functioning. Objective sleep metrics were assessed with the use of the Zmax EEG headband and the autoscoring algorithm of the Dreamento toolbox.^28^ The performance of the device and the algorithm has been validated before by comparison with polysomnography scored according to the standards of the American Academy of Sleep Medicine,^29^ showing a Cohen’s kappa of 72% compared to manual human scoring.^30^ For this study, sleep autoscoring was performed using a yet unpublished extension of the Dreamento toolbox, which identifies and excludes artefactual epochs. The objective sleep metrics included total sleep time (TST), sleep efficiency, sleep onset latency (SOL), wake after sleep onset (WASO), and proportions of rapid eye movement sleep and deep sleep. Subjective sleep quality and fatigue were measured with the Pittsburgh Sleep Quality Inventory and the Functional Assessment of Chronic Illness Therapy-Fatigue.

Stool samples were obtained to measure fecal calprotectin as a surrogate for bowel inflammation. Blood tests included hemoglobin, C-reactive protein, and albumin. If a participant reported a flare, the medical record was tracked. The start of a flare was defined as the date of change in IBD medication, elevated fecal calprotectin levels, or endoscopy with disease activity. Clinical disease activity was assessed by the clinician-administered instruments HBI for CD and SCCAI for UC. Included self-report measures were subjective disease control (IBD-control) and IBD-related QoL (Short Inflammatory Bowel Disease Questionnaire).

### Statistical Analysis

#### Sample size

Based on a meta-analysis of MBCT in IBD, we anticipated the effect size to be Cohen’s *d = *0.51.^31^ With an error probability of 0.05, 0.80 power, and 1:1 allocation ratio, a total sample size of 98 was required. Accounting for an averaged dropout rate of 28%, based on two similar RCTs in IBD,^32,33^ our corrected sample size was 136 (*n* = 68 per condition).

#### Treatment effects

All analyses were conducted in SPSS version 29 and performed primarily on the intention-to-treat (ITT) sample with sensitivity analyses on the per protocol (PP) sample. This was operationalized by having attended at least four sessions of MBCT in the intervention condition, and not having attended any mindfulness sessions in the TAU condition.

Treatment effects were analyzed using linear mixed effect models (LMMs). For post-intervention analyses, “group” (MBCT + TAU versus TAU alone), “time” (T0, T1), and “group*time” interaction effects were added as fixed effects, controlling for the stratification variables (ie, hospital, sex, and IBD type). For follow-up analyses, “group,” “time” (T1, T2, T3, T4), and “group*time” were added as fixed effects, controlling for the stratification variables and baseline scores. The variable “MBCT group” was not added as a random effect because the intra-cluster correlation was 0.005 (calculated for the HADS). Linear mixed effect models performed best when a random intercept for participants was added. Restricted maximum likelihood was used to obtain unbiased estimates of variance components in the mixed-effects model.

Effect sizes of the post-intervention treatment effects were calculated using Cohen’s *d*. Effect sizes of 0.20–0.50 were considered small, 0.50–0.80 medium, and > 0.80 large.

##### Objective sleep metrics

The raw EEG data of the baseline recordings were visually screened following the criteria of the Zmax validation study,^30^ requiring at least 75% of useful data and a minimum of 5.5 hours’ time in bed. Only participants with at least one night of sufficient quality were invited for the post-intervention assessment. For the definite selection of recordings with sufficient quality for analysis, two researchers independently reassessed raw EEG recordings. Recordings were excluded if: 

< 75% useful data, < 5.5 hours of time in bed, or if sleep stage scoring was inaccurate. Discrepancies were discussed until consensus was reached, with advice from an involved neuroscientist with expertise in sleep research (MD) if needed. Additionally, the sleep metrics were used to check whether the minimum useful data and time in bed were met. After this selection phase, means of the sleep scoring parameters were calculated per assessment. Logarithmic transformations were applied to SOL and WASO to acquire normality. Consequently, data were analyzed using the post-intervention LMM. Because of the exploratory nature of the analyses, we did not apply an adjustment for multiple comparisons.

##### Laboratory tests and flares

Logarithmic transformations were applied to C-reactive protein and fecal calprotectin due to their right-skewed distributions. Data were analyzed using the model most similar to the post-intervention LMM, incorporating T0, T2, and T4 for “time”. A Cox regression analysis was performed to analyze the differences between MBCT + TAU and TAU alone in time to flare within the 12-month study period. In retrospect, two participants were not in remission at the start of the study and were censored at baseline as having a flare.

#### Moderation analyses

Univariate moderation analyses were performed using Analysis of Covariance (ANCOVA), with the difference in scores for psychological distress (HADS) and well-being (MHC-SF) before and after the intervention as dependent variables. The model included “group,” “moderator,” and their interaction term to test for moderation. The following moderators were used: age, sex, level of education, IBD type, disease duration in years, medication use, repetitive negative thinking, mindfulness skills, self-compassion, psychological distress, and well-being.

#### Mediation analyses

Mediation analyses were performed to assess if mindfulness skills, self-compassion, repetitive negative thinking, and sleep quality (partially) explained the effect on psychological distress at post-intervention. The approach suggested by Preacher and Hayes for conducting multiple mediation models was used^34^ and performed in the SPSS PROCESS macro version 4.2.

## Results

### Recruitment and Adherence

**Figure 1.**
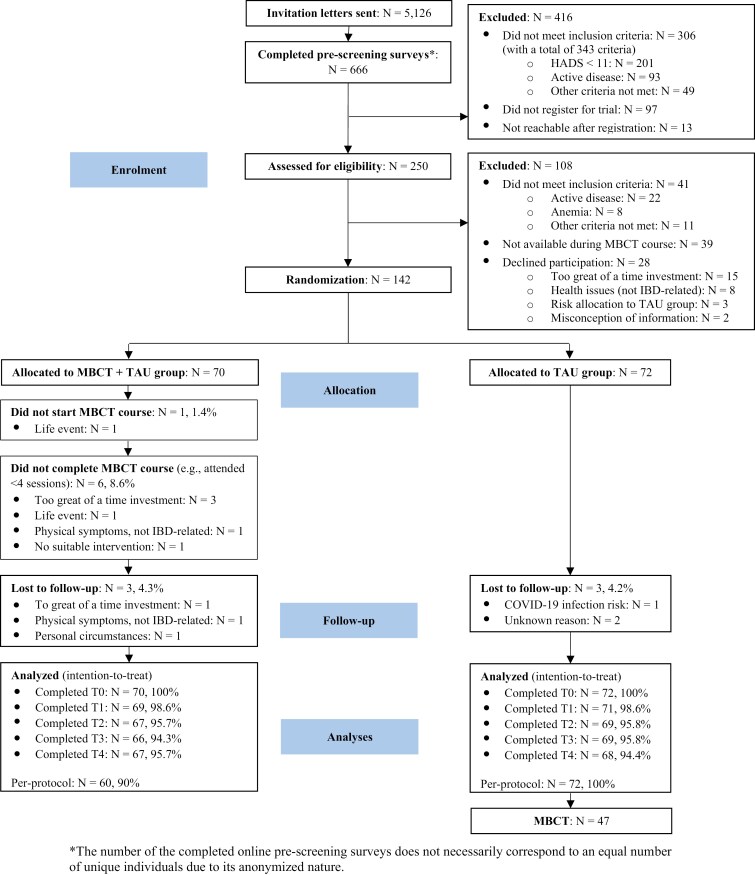
**CONSORT diagram for the MindIBD trial**. HADS, Hospital Anxiety and Depression Scale; IBD, Inflammatory Bowel Disease; MBCT, Mindfulness-Based-Cognitive Therapy; TAU, treatment as usual. *The number of the completed online pre-screening surveys does not necessarily correspond to an equal number of unique individuals due to its anonymized nature.

Figure 1 shows the CONSORT diagram of the study. An invitation letter was sent to 5126 IBD patients. The online prescreening survey was completed 666 times, and interviews were conducted with 250 (4.9%) patients. Of these, 142 participants were randomly allocated to the MBCT + TAU group (*n* = 70) or TAU alone group (*n* = 72). During follow-up, 6 participants (4.2%), equally divided between both groups, dropped out during follow-up. Two of the dropouts in the MBCT + TAU group also did not complete the MBCT course (ie, attended less than four sessions).

Within the MBCT + TAU group, the median number of attended sessions was 7 [IQR: 6–8]. Seven (10.0%) participants did not complete the MBCT course. The non-completers more frequently had a low or medium education level (*n* = 6/7,85.7%) than the completers (*n* = 25/63, 39.7%; Fisher’s exact test *P = *.039). There were no significant differences regarding the other demographics, disease characteristics, and baseline HADS score.

### Study Population

There were no significant differences in demographic and disease-specific characteristics between the two groups, as shown in Table 1. Engagement in psychological treatments other than MBCT, both prior to and during the follow-up period, was also comparable.

**Table 1.** Demographic and disease characteristics of the study population.

	Total *n* = 142	MBCT + TAU *n* = 70	TAU alone *n* = 72
Female, *n* (%)	91 (64.1)	45 (64.3)	46 (63.9)
Age, μ* ± SD*	48.6 ± 14.0	49.1 ± 15.4	48.2 ± 12.6
Marital status, *n* (%)			
Married or living together	101 (71.1)	48 (68.6)	53 (73.6)
Long-distance relationship	8 (5.6)	3 (4.3)	5 (6.9)
Single, divorced, or widow(er)	33 (23.2)	19 (27.1)	14 (19.4)
Education level^a^, *n* (%)			
Lower	13 (9.2)	6 (8.6)	7 (9.7)
Medium	53 (37.3)	25 (35.7)	28 (38.9)
Higher	76 (53.5)	39 (55.7)	37 (51.3)
Working, *n* (%)	98 (69.0)	48 (68.6)	50 (69.4)
Hospital, *n* (%)			
Radboudumc^b^	24 (16.9)	10 (14.3)	14 (19.4)
Jeroen Bosch Hospital^c^	40 (28.2)	21 (30.0)	19 (26.4)
Canisius Wilhelmina Hospital^c^	30 (21.1)	15 (21.4)	15 (20.8)
Rijnstate Hospital^c^	48 (33.8)	24 (34.3)	24 (33.3)
IBD-type, *n* (%)			
Crohn’s disease	68 (47.9)	34 (48.6)	34 (47.2)
Ulcerative colitis	74 (52.1)	36 (51.4)	38 (52.8)
Disease duration, *median [IQR]*	12.0 [8.0–23.0]	13.5 [7.0–27.3]	12.0 [8.0–22.0]
Stoma, *n* (%)	7 (5.1)	3 (4.3)	4 (5.6)
Drugs, *n* (%)			
None	38 (26.8)	17 (24.3)	21 (29.2)
Topical therapy/5-ASA	56 (39.4)	30 (42.9)	26 (36.1)
Immunosuppressive therapy^d^	39 (27.5)	19 (27.1)	20 (27.8)
Biologics and small molecules	37 (26.1)	18 (25.7)	19 (26.4)
Psychological treatment^e^, *n* (%)			
At baseline	30 (21.1)	15 (21.4)	15 (20.8)
During follow-up	14 (9.9)	8 (11.4)	6 (8.3)

^a^Based on International Standard Classification of Education: lower education includes the primary education plus the first three years of the upper secondary education (HAVO/VWO), prevocational secondary education (VMBO), and assistant’s training (MBO-1); medium education includes upper secondary education (HAVO/VWO), (basic) vocational training (MBO-2/-3), and middle management and specialist education (MBO-4); higher education includes associate-, Bachelor-, Master-, and doctoral degree programs (HBO/WO).

^b^University hospital.

^c^General hospital.

^d^Immunosuppressive therapy includes steroids, thiopurines, methotrexate, and calcineurin inhibitors.

^e^Other than mindfulness, including psychologist (*n* = 31), mental health nurse practitioner at GP office (*n* = 5), rehabilitation program (*n* = 3), medical social worker (*n* = 3), psychiatrist (*n* = 2).

Abbreviations: 5-ASA, 5-aminosalicylic acid; IBD, inflammatory bowel disease; MBCT, Mindfulness-Based Cognitive Therapy; TAU, treatment as usual.

### Post-Intervention Treatment Effects

Mean scores for the primary and secondary outcomes at baseline and post-intervention are shown in Tables 2 and 3, along with ITT analyses of the post-intervention treatment effects. Per protocol analyses are added as Table S2.

**Table 2.** Post-intervention treatment effects of MBCT on self-report questionnaires.

Outcome	Group	Baseline (T0)		Post-interv. (T1)		Group × Time^a^	
		Mean ± SD *n* = 142		Mean ± SD *n* = 140		Unstandardized coefficient B (95% confidence interval)	*P* value^b^
*Primary outcome: psychological distress*							
Psychological distress (HADS-Total)	MBCT + TAU	16.4 ± 7.7		11.6 ± 6.5		−3.4 (−5.5 to −1.6)	<.001*
	TAU alone	16.3 ± 6.2		15.1 ± 7.3			
Anxiety symptoms (HADS-A)	MBCT + TAU	9.2 ± 4.2		7.0 ± 3.5		−1.6 (−2.7 to −0.5)	.004*
	TAU alone	9.0 ± 3.2		8.4 ± 3.9			
Depressive symptoms (HADS-D)	MBCT + TAU	7.2 ± 4.2		4.6 ± 3.6		−2.0 (−3.0 to −0.9)	<.001*
	TAU alone	7.2 ± 3.7		6.7 ± 4.0			
*Other mental health-related measures*							
Repetitive negative thinking (PTQ)	MBCT + TAU	29.9 ± 12.5		26.6 ± 11.4		−2.6 (-5.6 to 0.4)	.086
	TAU alone	28.5 ± 11.0		27.7 ± 11.5			
Mindfulness skills (FFMQ-SF)	MBCT + TAU	75.2 ± 9.3		81.0 ± 10.2		3.8 (1.0 to 6.6)	.007*
	TAU alone	74.6 ± 10.5		76.5 ± 12.2			
Self-compassion (SCS-SF)	MBCT + TAU	45.7 ± 12.9		52.2 ± 12.2		3.7 (0.7 to 6.7)	.015*
	TAU alone	47.8 ± 13.6		50.6 ± 14.8			
Well-being (MHC-SF)	MBCT + TAU	2.6 ± 1.1		2.9 ± 0.9		0.3 (0.04 to 0.5)	.022*
	TAU alone	2.7 ± 0.8		2.7 ± 0.9			
*Sleep quality and fatigue*							
Sleep quality (PSQI)	MBCT + TAU	7.9 ± 3.4		6.8 ± 3.2		−0.7 (−1.6 to 0.1)	.096
	TAU alone	7.6 ± 3.8		7.2 ± 3.5			
Fatigue (FACIT-F, fatigue subscale)	MBCT + TAU	30.2 ± 10.2		34.3 ± 10.1		2.4 (−0.05 to 4.8)	.055
	TAU alone	28.7 ± 11.2		30.6 ± 11.3			
*IBD-related measures*							
Clinical index for disease activity in Crohn’s disease (HBI), *n* = 68	MBCT + TAU		2.4 ± 2.0		2.4 ± 2.1	−0.9 (−2.2 to 0.4)	.162
	TAU alone		2.4 ± 1.8		3.3 ± 3.4		
Clinical index for disease activity in ulcerative colitis (SCCAI), *n* = 72	MBCT + TAU		1.0 ± 1.0		1.4 ± 1.9	0.6 (−0.3 to 1.5)	.165
	TAU alone		1.2 ± 1.3		1.0 ± 1.3		
Disease control (IBD-Control)	MBCT + TAU	12.1 ± 3.2		12.3 ± 3.5		−0.7 (−1.8 to 0.4)	.222
	TAU alone	12.2 ± 3.1		13.0 ± 3.2			
IBD-related quality of life (SIBDQ)	MBCT + TAU	50.2 ± 6.1		51.0 ± 7.0		1.1 (−0.83 to 3.39)	.262
	TAU alone	51.6 ± 6.9		51.3 ± 7.5			

^a^TAU alone is the reference category.

^b^Bold text plus an asterisk denotes statistical significance (*P* < .05).

Abbreviations: FACIT-F, Functional Assessment of Chronic Illness Therapy-Fatigue; FFMQ-SF, Five Facet Mindfulness Questionnaire-Short Form; HADS, Hospital Anxiety and Depression Scale; HBI, Harvey-Bradshaw Index; IBD, Inflammatory Bowel Diseases; MBCT, Mindfulness-Based Cognitive Therapy; MHC-SF, Mental Health Continuum-Short Form; PSQI, Pittsburgh Sleep Quality Inventory; PTQ, Perseverative Thinking Questionnaire; SIBDQ, Short Inflammatory Bowel Disease Questionnaire; SCCAI, Simple Clinical Colitis Activity Index; SCS-SF, Self-Compassion Scale-Short Form; TAU, treatment as usual.

**Table 3.** Post-intervention treatment effects of MBCT on objective sleep metrics.

Outcome	Group	Baseline (T0)	Post-intervention (T1)	Group × Time^a^	
		Mean ± SD *n* = 70	Mean ± SD *n* = 56	Unstandardized coefficient B (95% confidence interval)	*P* value^b^
Total sleep time (min)	MBCT + TAU	401.4 ± 59.6	374.7 ± 65.3	−38.0 (−67.5 to −8.5)	.013*
	TAU alone	382.6 ± 54.5	396.9 ± 54.6		
Sleep efficiency (%)	MBCT + TAU	80.4 ± 7.4	77.3 ± 11.3	-−3.2 (−8.6 to 2.2)	.234
	TAU alone	80.7 ± 9.7	82.3 ± 9.2		
Sleep onset latency (min)	MBCT + TAU	14.3 ± 9.9	19.9 ± 31.1	*Log:* 0.04 (−0.1 to 0.2)	.582
	TAU alone	9.4 ± 4.6	9.1 ± 5.1		
Wake after sleep onset (min)	MBCT + TAU	68.8 ± 31.7	72.4 ± 32.0	*Log:* 0.005 (−0.1 to 0.1)	.929
	TAU alone	65.5 ± 40.5	61.1 ± 28.3		
Proportion of REM sleep (%)	MBCT + TAU	23.9 ± 7.2	23.6 ± 7.9	−1.3 (−4.1 to 1.6)	.373
	TAU alone	27.0 ± 7.1	27.5 ± 8.1		
Proportion of deep sleep (%)	MBCT + TAU	34.6 ± 13.3	37.5 ± 15.6	5.0 (1.0 to 9.0)	.016*
	TAU alone	35.0 ± 12.1	32.5 ± 10.6		

^a^TAU alone is reference category.

^b^Bold text plus an asterisk denotes statistical significance.

Abbreviations: MBCT, Mindfulness-Based Cognitive Therapy; REM, rapid eye movement; TAU, treatment as usual.

Participants in the MBCT + TAU group reported less psychological distress at post-intervention than participants in the TAU alone group, with a medium effect size (*d* = −0.61). Per protocol analysis showed a more pronounced effect (*d* = −0.74).

Participants in the MBCT + TAU group also developed more mindfulness skills (*d* = 0.46) and self-compassion (*d* = 0.42). Additionally, the MBCT + TAU group showed more improvement in well-being (*d* = 0.41).

Due to logistic problems, the EEG headbands were not available for the first two (of nine) groups. Hundred participants completed the baseline assessment with the EEG headband, of whom 70 participants provided at least one quality recording (MBCT + TAU *n* = 34, TAU alone *n* = 36). Post-intervention, 86 participants completed the assessment, resulting in 56 measurements of sufficient quality (MBCT + TAU *n* = 27, TAU alone *n* = 29). The median of unuseful data was 1.4% [IQR: 0.5–4.9] at baseline and 1.7% [IQR: 0.5–5.2] at post-intervention, with no differences between the groups.

Participants in the MBCT + TAU group showed a significant reduction in TST (*d* = −0.67) with an increase of the deep sleep proportion compared to the TAU alone group (*d* = 0.70). In the subjective assessment of sleep quality, we observed a small but non-significant effect in favor of MBCT (*d* = −0.28). Additionally, participants in the MBCT + TAU group reported less fatigue, which was statistically significant in the PP analysis (*d* = 0.41) but not in the ITT analysis (*d* = 0.33).

### Follow-Up Treatment Effects

Mean scores of the follow-up assessments and the results of the corresponding ITT analyses are shown in Table 4. Results of laboratory tests are presented in Table 5. Additionally, PP analyses are added as Table S2. HADS scores in the MBCT + TAU group remained stable during follow-up, indicating a consolidation of the post-intervention treatment effect. However, the trend toward statistical significance (p = .092) in between group comparisons can be attributed to a decline in the HADS scores in the TAU alone group over time. Despite this decline, scores in the TAU alone group remained highger than those in the MBCT + TAU group. 

**Table 4.** Follow-up treatment effects of MBCT on self-report questionnaires.

Outcome	Group	T1	T2	T3	T4	Group × Time^a^	
		Mean ± SD *n* = 140	Mean ± SD *n* = 136	Mean ± SD *n* = 135	Mean ± SD *n* = 135	Unstandardized coefficient B (95% confidence interval)	*P* value^b^
*Primary outcome: psychological distress*							
Psychological distress (HADS-Total)	MBCT + TAU	11.6 ± 6.5	12.8 ± 6.9	11.9 ± 7.2	11.1 ± 7.0	0.5 (−0.1 to 1.1)	.092
	TAU alone	15.1 ± 7.3	13.6 ± 6.4	13.8 ± 7.6	12.8 ± 7.9		
Anxiety symptoms (HADS-A)	MBCT + TAU	7.0 ± 3.5	7.4 ± 3.8	7.0 ± 4.0	6.5 ± 3.9	0.2 (−0.2 to 0.5)	.321
	TAU alone	8.4 ± 3.9	7.6 ± 3.5	7.9 ± 4.1	7.3 ± 2.1		
Depressive symptoms (HADS-D)	MBCT + TAU	4.6 ± 3.6	5.4 ± 3.9	5.0 ± 3.7	4.6 ± 3.6	0.3 (0.008 to 0.7)	**.045**
	TAU alone	6.7 ± 4.0	6.0 ± 3.6	5.9 ± 4.1	5.5 ± 4.4		
*Other mental health-related measures*							
Repetitive negative thinking (PTQ)	MBCT + TAU	26.6 ± 11.4	24.5 ± 11.8	23.8 ± 12.5	22.6 ± 10.9	−0.3 (−1.2 to 0.6)	.480
	TAU alone	27.7 ± 11.5	25.9 ± 12.0	25.3 ± 11.5	25.5 ± 11.2		
Mindfulness skills (FFMQ-SF)	MBCT + TAU	81.0 ± 10.2	81.0 ± 10.8	81.0 ± 12.1	82.6 ± 11.8	−0.5 (−1.2 to 0.3)	.251
	TAU alone	76.5 ± 12.2	78.7 ± 12.5	79.1 ± 13.6	79.6 ± 13.0		
Self-compassion (SCS-SF)	MBCT + TAU	52.2 ± 12.2	50.3 ± 13.2	51.9 ± 13.6	54.1 ± 13.9	0.9 (0.08 to –1.8)	**.032**
	TAU alone	50.6 ± 14.8	51.9 ± 13.7	51.3 ± 13.7	50.5 ± 15.6		
Well-being (MHC-SF)	MBCT + TAU	2.9 ± 0.9	2.9 ± 0.9	2.9 ± 1.0	3.1 ± 1.0	−0.003 (−0.07 to 0.06)	.923
	TAU alone	2.7 ± 0.9	2.9 ± 0.8	2.9 ± 1.0	2.9 ± 0.9		
*Sleep quality (subjective) and fatigue*							
Sleep quality (PSQI)	MBCT + TAU	6.8 ± 3.2	6.5 ± 3.2	6.6 ± 3.6	6.6 ± 3.6	0.2 (−0.1 to 0.5)	.288
	TAU alone	7.2 ± 3.5	7.0 ± 3.6	6.8 ± 3.8	6.7 ± 4.0		
Fatigue (FACIT-F, fatigue subscale)	MBCT + TAU	34.3 ± 10.1	33.9 ± 10.1	34.9 ± 10.2	34.8 ± 11.2	−0.7 (−1.5 to 0.1)	.093
	TAU alone	30.6 ± 11.3	31.1 ± 12.1	32.4 ± 11.4	32.7 ± 12.4		
*IBD-related measures*							
Clinical index for disease activity in Crohn’s disease (HBI), *n* = 64	MBCT + TAU	2.4 ± 2.1	2.5 ± 2.1	2.1 ± 1.8	2.3 ± 2.1	0.2 (−0.2 to 0.6)	.264
	TAU alone	3.3 ± 3.4	2.3 ± 1.9	2.9 ± 2.5	2.3 ± 2.0		
Clinical index for disease activity in ulcerative colitis (SCCAI), *n* = 70	MBCT + TAU	1.4 ± 1.9	1.1 ± 1.7	1.0 ± 1.3	1.3 ± 2.2	−0.07 (−0.3 to 0.2)	.594
	TAU alone	1.0 ± 1.3	1.3 ± 1.5	1.1 ± 1.4	1.2 ± 1.8		
Disease control (IBD-Control)	MBCT + TAU	12.3 ± 3.5	12.3 ± 3.5	12.7 ± 3.5	12.5 ± 4.0	0.2 (−1.2 to 0.6)	.232
	TAU alone	13.0 ± 3.2	12.7 ± 3.3	12.4 ± 3.7	12.5 ± 3.7		
IBD-related quality of life (SIBDQ)	MBCT + TAU	51.0 ± 7.0	51.5 ± 6.9	51.3 ± 6.4	52.0 ± 7.6	0.5 (−0.2 to 1.2)	.181
	TAU alone	51.3 ± 7.5	51.4 ± 7.2	51.3 ± 7.4	50.3 ± 8.7		

^a^TAU alone is the reference category.

^b^ *P* ≥ .05 indicates consolidation of the treatment effect; *P* < .05 indicates a differential change between the groups during follow-up.

Abbreviations: FACIT-F, Functional Assessment of Chronic Illness Therapy-Fatigue; FFMQ-SF, Five Facet Mindfulness Questionnaire-Short Form; HADS, Hospital Anxiety and Depression Scale; HBI, Harvey-Bradshaw Index; IBD, Inflammatory Bowel Diseases; MBCT, Mindfulness-Based Cognitive Therapy; MHC-SF, Mental Health Continuum-Short Form; PSQI, Pittsburgh Sleep Quality Inventory; PTQ, Perseverative Thinking Questionnaire; SIBDQ, Short Inflammatory Bowel Disease Questionnaire; SCCAI, Simple Clinical Colitis Activity Index; SCS-SF, Self-Compassion Scale-Short Form; TAU, treatment as usual; T1, 3 months after baseline; T2, 6 months after baseline; T3, 9 months after baseline; T4, 12 months after baseline.

**Table 5.** Follow-up treatment effects of MBCT on laboratory tests.

Outcome	Group	T0		T2		T4		Group × Time^a^	
		*n*	Mean ± SD	*n*	Mean ± SD	*n*	Mean ± SD	Unstandardized coefficient B (95% confidence interval)	*P* value^b^
Fecal calprotectin (μg/g)	MBCT + TAU	70	83.3 ± 228.0	65	120.8 ± 365.1	65	57.9 ± 112.8	*Log:* −0.05 (−0.09 to −0.01)	.010*
	TAU alone	70	53.9 ± 60.4	64	61.8 ± 104.4	65	83.1 ± 121.6		
Hemoglobin (mmol/L)	MBCT + TAU	70	8.6 ± 0.8	64	8.6 ± 0.9	66	8.6 ± 0.8	−0.03 (−0.06 to 0.01)	.174
	TAU alone	72	8.6 ± 0.6	66	8.6 ± 0.7	69	8.7 ± 0.7		
C-reactive protein (mg/L)	MBCT + TAU	69	3.7 ± 3.1	64	5.0 ± 7.5	65	3.9 ± 2.9	*Log:* −0.007 (−0.03 to 0.02)	.514
	TAU alone	72	4.0 ± 4.3	66	3.7 ± 3.0	69	5.5 ± 8.8		
Albumin (g/L)	MBCT + TAU	64	40.6 ± 3.9	58	39.3 ± 7.9	60	40.8 ± 3.3	0.3 (−0.3 to 0.8)	.349
	TAU alone	69	40.6 ± 3.5	61	40.9 ± 3.6	66	39.6 ± 7.4		

^a^TAU alone is the reference category.

^b^Bold text plus an asterisk denotes statistical significance.

Abbreviations: MBCT, Mindfulness-Based-Cognitive Therapy; TAU, treatment as usual, T0, baseline; T2, 6 months after baseline; T4, 12 months after baseline.

The effect on self-compassion in the MBCT + TAU group increased over the follow-up period (*P* = .032), although this effect was not statistically significant in the PP analysis (*P* = .070). For the other self-reported outcomes, consolidation of the post-intervention treatment effect was seen.

Fecal calprotectin levels of participants in the MBCT + TAU group showed an increase between baseline and 6 months follow-up, followed by a decrease at 12 months follow-up. Compared to the control group, the fecal calprotectin level in the MBCT + TAU group showed a significant decrease in fecal calprotectin over time (*d* = −0.49). No differences were observed in the occurrence of flares (MBCT + TAU: 10 (14.3%) versus TAU alone: 12 (16.7%); Cox regression *P* = .829), nor in changes of medication regimes (MBCT + TAU: 7 (10.0%) versus TAU alone: 9 (12.5%); χ^2^  *P* = .837).

### Moderation Analyses

Moderation analysis results are presented in Table S3. Higher psychological distress at baseline was associated with a stronger effect of MBCT on psychological distress (B (95% CI, −0.3 (−0.6 to −0.06), *P* = .016). Additionally, longer disease duration was associated with a stronger effect of MBCT on well-being (B (95% CI, 0.03 (0.008 to 0.047), *P* = .005).

### Mediation Analyses

Mediation analysis results are presented in Table S4. Based on the univariate analyses, the effect of MBCT on psychological distress post-intervention were partially mediated by mindfulness skills (β (95% CI, −0.2 (−0.3 to −0.05), *P* < .05) and self-compassion (β (95% CI, −0.08 (−0.2 to −0.005), *P* < .05). When performing the multivariate analysis, only mindfulness skills had an independent mediating effect on psychological distress (β (95% CI, −0.1 (−0.3 to −0.03), *P* < .05).

## Discussion

The aim of the MindIBD trial was to evaluate the effectiveness of MBCT in patients with IBD. In the present study, we demonstrate that MBCT resulted in a stronger reduction of psychological distress post-intervention compared to TAU alone, with a consolidation of the effect during one-year follow-up. Mindfulness-Based Cognitive Therapy also positively affected the development of mindfulness skills, self-compassion, and well-being. Developing mindfulness skills (partially) explained the effect on psychological distress post-intervention. Subjective reports of sleep and fatigue showed a trend toward improvement following MBCT. On a biological level, we observed a reduction in total sleep time after MBCT with an increase in the proportion of deep sleep. Finally, MBCT appeared to have a positive effect on fecal calprotectin levels at 12-month follow-up, although our study did not observe a reduction in flares.

To the best of our knowledge, this study is the largest RCT investigating the effectiveness of MBCT in patients with IBD, and the first with psychological distress (ie, symptoms of both anxiety and depression) as the primary outcome in an adult IBD population. The positive results on psychological distress align with available meta-analyses, including patients with somatic conditions,^35^ such as multiple sclerosis^36^ and cancer.^37^ Moreover, this well-powered study is an encouraging addition to the existing contradictory literature in IBD populations.^13,14^ One of the reasons for these variable results might have been the heterogeneity of the interventions. Nevertheless, inconsistencies in results persisted among the studies that used MBSR or MBCT (k = 6) and could therefore possibly be explained by small sample sizes (*n* = 43–64)^15,32,33,38,39^ and/or high dropout rates (30%-44%).^18,33^

Interestingly, our study did not show improvement in the IBD-related questionnaires, which mainly concern the absence or presence of bowel symptoms. Even though MBCT does not affect these symptoms, individuals who completed MBCT are probably better able to cope with bowel symptoms or during flares due to the mindfulness skills they have developed, such as non-judging and non-reactivity. This idea is supported by the study of Jedel et al., which reported a better QoL during a flare after completing MBSR.^32^

Despite its exploratory nature, the observed increase in the proportion of deep sleep following MBCT is an interesting finding. Deep sleep is of particular interest in IBD because of its well-established role in immunological processes: pro-inflammatory cytokines such as IL-1, IL-6, and TNF-α are implicated in poor sleep and specifically disrupt deep sleep, and are also involved in the pathogenesis of IBD.^40,41^ Therefore, optimizing deep sleep could positively affect the disease course of IBD.^42^ Besides, there is some evidence that long-term meditation practice is associated with improved deep sleep.^43^

Finally, over the study period, we observed a decrease in fecal calprotectin levels. While the evidence for brain-to-gut effects (ie, the detrimental impact of psychological distress on disease outcomes in IBD) is convincing,^6^ the support for interventions that improve psychological symptoms leading to more stable disease outcomes is not.^44^ The existing literature on the impact of mindfulness on fecal calprotectin is mixed.^14^ However, a recent meta-analysis by Naude et al. found a positive effect of mindfulness on fecal calprotectin after conducting a sensitivity analysis to reduce heterogeneity.^14^ In our study, we first observed an increase in fecal calprotectin levels, followed by decrease, suggesting that longer follow-up may be necessary to fully capture the effects of MBCT on disease outcomes.

### Strengths and Limitations

An important strength of our study is the combination of objective outcome measures (ie, fecal calprotectin and objective sleep metrics) and validated self-report questionnaires. Additionally, by inviting all IBD patients of the participating hospitals (both university and general hospitals), we recruited a sample that is as representative as possible and minimized the risk of a selection bias on the part of the clinician, ie, “gate keeping.” Even though the study was conducted during the COVID-19 pandemic, recruitment was relatively easy and we had low dropout numbers. The ease of recruitment suggests a need for psychological interventions such as MBCT. High retention rates may be explained by a committed study population due to the recruitment strategy, qualified MBCT teachers having experience with both psychiatric and somatic populations, and the close involvement of the research team. Finally, we would like to emphasize our multidisciplinary approach, involving gastroenterologists, experienced mindfulness teachers with a background in psychiatry and psychology, a neuroscientist committed to sleep research, the patient association, and an IBD patient representative.

A methodological limitation is that blinding was not possible for both participants and the coordinating researcher, whose active involvement during data collection was limited to the clinician-administered interviews. Hence, we cannot rule out expectation effects, especially given the use of self-report questionnaires. Besides that, although the mindfulness teachers involved had ample experience with medical populations, their competence levels based on the MBI:TAC evaluation varied between advanced beginner and proficient.^23^ Given that the intra-cluster correlation coefficient for MBCT groups was < 0.05, the differences in competence might not have had too much influence on the effect of MBCT. This aligns with findings from a previous study in patients with recurrent depression.^45^In addition, the limitations of the objective sleep assessment should be acknowledged. First, we have not assessed other sleep disorders, such as obstructive sleep apnea, narcolepsy, or restless legs syndrome, that may have influenced the sleep metrics. Second, although wearing the EEG headband seemed feasible beforehand, we faced several logistical and technical issues during the study: the headband was not available for the first two groups, a significant number of nights were unusable due to problems with self-application or artifacts. In addition, although the algorithm for sleep stage scoring was validated with polysomnography,^30^ it had not yet been fully optimized or validated in patient groups. This field of research is in its early stages. Future developments are expected to considerably improve assessment quality.

### Research Implications

In this study, we made an initial attempt to evaluate the mechanisms of mindfulness in this population. Future research with intermediate assessments might help clarify the temporal sequence of the observed association between mindfulness skills and psychological distress. Moreover, mediation analyses exploring how mindfulness leads to reduced fecal calprotectin levels may offer valuable insights for refining the intervention to maximize its effectiveness on the disease course.

To our knowledge, this is the first study using EEG to objectively assess the effect of MBCT on sleep.^12^ Three previous studies in non-IBD populations used actigraphy which could not distinguish different sleep stages,^46–48^ something particulary relevant in inflammatory diseases such as IBD. Although the EEG headband may be a useful and cost-effective tool for assessments in home-based settings, many technical issues were encountered with the headband and the algorithm. Future research is needed to address these challenges and to replicate this study to validate these initial findings.

While a decrease in fecal calprotectin suggests MBCT may impact disease course, our study did not show a difference in flare occurrence. This could be attributed to the inactive disease of the participants at baseline and the low flare rate during the 1-year follow-up. We recommend a longer follow-up study to further investigate this.

### Clinical Implications

Our results demonstrate that MBCT could be a valuable addition to the currently limited number of psychological treatment options for patients with IBD with psychological distress. The group format of MBCT allows for the simultaneous treatment of more patients, potentially making it more cost-effective than individual therapy. Additionally, group therapy provides opportunities for peer support. However, due to logistical and financial barriers, the organization of group-delivered MBCT may not be easy in all settings, in which case individual MBCT could be considered. Our research group is currently conducting studies on cost-effectiveness, acceptability and feasibility, and implementation strategies to increase the accessibility of MBCT in the clinical care for IBD patients.

Additionally, there are several other important implications for clinical practice to consider. First, both the level of psychological distress and disease duration were moderators. This is possibly linked to a higher willingness and commitment, which is not surprising given the time and effort the training demands from participants. Given the notably higher proportion of lower-educated individuals among MBCT dropouts in our study, there is a need for strategies to enhance retention within these patients. Finally, while the effects of MBCT are maintained throughout the one-year follow-up—unlike the gradual reduction in effectiveness often seen with psychological interventions—our population still experiences significant levels of psychological distress after MBCT (HADS-total: 11.6 ± 6.5). Extending the intervention through booster sessions or follow-up (compassion) training could possibly lead to improved effectiveness.

## Conclusion

In conclusion, our study shows that group-delivered MBCT has the potential to improve psychological distress and well-being in IBD patients with elevated levels of psychological distress. Moreover, this study provides evidence for its possible impact on biological processes, such as sleep and inflammation. These promising findings set the stage for further investigation of the robustness of MBCT’s effectiveness in IBD, its working mechanisms, and the possibilities for large-scale implementation. Our findings may also serve as a basis for further investigating MBCT for other chronic inflammatory conditions, such as rheumatoid arthritis and multiple sclerosis.

## Supplementary Material

izaf116_Supplementary_Tables_S1-S4
