# Supplementary material for: Group-Delivered Mindfulness-Based Cognitive Therapy to Reduce Psychological Distress and Improve Sleep in Patients With Inflammatory Bowel Diseases: A Multicenter Randomized Controlled Trial (MindIBD)
Source: Inflamm Bowel Dis. 2025 Jul 14;31(11):3021–32. doi: 10.1093/ibd/izaf116 (PMC12638053; doi:10.1093/ibd/izaf116)
Supplement: izaf116_Supplementary_Tables_S1-S4 [file izaf116_supplementary_tables_s1-s4.docx]

**SUPPLEMENTARY MATERIAL**

**Supplementary table S1: Mindfulness-Based Cognitive Therapy program for IBD patients**

| Week: theme | Content | Meditation exercise | Homework |
| --- | --- | --- | --- |
| 1: The automatic pilot | Introduction on training and ground rules | Intention meditation | Body scan |
|  | Proposal round | Body scan | Eating mindfully |
|  | Experiential exercise: Raisin exercise | Attention for the breath | Daily routine activity with attention |
|  | Psycho-education: how mindfulness can help for physical and mental problems in IBD |  |  |
| 2: Seeing clearly - thoughts are not facts | Observation exercise: window watching | Body scan | Body scan |
|  | Interpretation exercise: walking down the street | Sitting meditation (focus on breath) | Attention for the breath (5-10 min) |
|  |  |  | Daily routine activity with attention (new activity) |
|  |  |  | Complete calendar pleasant experiences |
| 3: From doing to being | Reflection on positive experiences, including psycho-education: triangle of awareness | Sitting meditation (focus on breath and body sensations) | Alternating sitting meditation with lying movement exercises |
|  |  | Movement exercises (lying) | 3-minute breathing space (fixed times) |
|  |  | 3-minute breathing space (introduction) | Daily routine activity with attention (new activity) |
|  |  |  | Complete calendar unpleasant experiences |
| 4: Stay present and allow what is there | Reflection on negative experiences, including psycho-education: connection to automatic reactions | Sitting meditation (total version) | Alternating sitting meditation with lying movement exercises or walking meditation |
|  | Psycho-education: automatic thoughts (questionnaire) | 3-minute breathing space (coping) | 3-minute breathing space (fixed times and difficult situations) |
|  |  | Walking meditation | Complete calendar stressful experiences |
| 5: Creating space to make choices | Halfway reflection of the training | Sitting meditation (with a difficulty) | Alternating sitting meditation with movement exercises or body scan |
|  | Reflection on how to respond to stress, including psycho-education: reaction vs. response | 3-minute breathing space | 3-minute breathing space (fixed times and difficult situations) |
|  |  | Movement exercises (standing) | Being aware of (automatic) reactions to stress, practicing responding in difficult situations, and exploring alternative ways of responding |
|  |  |  | Complete calendar difficult communication |
| 6: Communicating with awareness | Reflection on communication diary with exercise in mindful communication | Sitting meditation | Alternating sitting meditation with movement exercises or body scan (without audio files) |
|  | Psycho-education: energy givers and takers | Movement exercises (standing) | 3-minute breathing space (difficult situations) |
|  |  | 3-minute breathing space | Mindful communication with someone close |
|  |  |  | Paying attention to recognizing own patterns in communication |
|  |  |  | Energy balance and action plan |
| Silent day | Introduction of silent day and ground rules | Movement exercises (lying, standing) |  |
|  | Reflection on experiences during silent day | Sitting meditation |  |
|  |  | Walking meditation (inside, outside) |  |
|  |  | Loving kindness meditation (3 steps) |  |
|  |  | Mountain meditation |  |
| 7: Taking care of yourself | Reflection on practice without guidance | Movement exercises (in pairs) | Each day an exercise of own choice |
|  | Reflection on silent day | Sitting meditation (choiceless awareness, without guidance) | Practice daily activities with attention |
|  | Reflection on action plan | 3-minute breathing space | Prepare self-evaluation |
| 8: The 8^th^ week continues for the rest of your life | Evaluation of the training | Body scan |  |
|  | How to continue practicing mindfulness in your daily life | Sitting meditation (5 words to remember mindfulness) |  |
|  |  | 3-minute breathing space |  |

**Supplementary table S2: per protocol analyses of treatment effects**

| Outcome | Model | Group*time^a^ |  |
| --- | --- | --- | --- |
|  |  | *Unstandardized coefficient B (95% CI)* | *P value^b^* |
| *Primary outcome: psychological distress* | | | |
| Psychological distress (HADS-Total) | Post-intervention | -4.2 (-6.1 – -2.3) | **<0.001*** |
|  | Follow-up | 0.6 (-0.03 – 1.2) | 0.064 |
| Anxiety symptoms (HADS-A) | Post-intervention | -2.0 (-3.0 – -0.9) | **<0.001*** |
|  | Follow-up | 0.2 (-0.1 – 0.6) | 0.188 |
| Depressive symptoms (HADS-D) | Post-intervention | -2.2 (-3.3 – -1.1) | **<0.001*** |
|  | Follow-up | 0.3 (0.01 – 0.7) | **0.047** |
| *Other mental health-related measures* | | | |
| Repetitive negative thinking (PTQ) | Post-intervention | -2.8 (-5.8 – 0.3) | 0.074 |
|  | Follow-up | -0.3 (-1.2 – 0.6) | **0.553** |
| Mindfulness skills (FFMQ-SF) | Post-intervention | 4.2 (1.3 – 7.0) | **0.005*** |
|  | Follow-up | -0.5 (-1.3 – 0.3) | 0.228 |
| Self-compassion (SCS-SF) | Post-intervention | 3.9 (0.8 – 7.0) | **0.014*** |
|  | Follow-up | 0.8 (-0.07 – 1.7) | 0.070 |
| Well-being (MHC-SF) | Post-intervention | 0.3 (0.05 – 0.5) | **0.019*** |
|  | Follow-up | -0.007 (-0.07 – 0.6) | 0.828 |
| *Sleep and fatigue* | | | |
| EEG – total sleep time | Post-intervention | -36.1 (-65.3 – -6.9) | **0.016*** |
| EEG – sleep efficiency | Post-intervention | -3.0 (-8.3 – 2.4) | 0.276 |
| EEG – sleep onset latency (log) | Post-intervention | 0.04 (-0.1 – 0.2) | 0.649 |
| EEG – wake after sleep onset (log) | Post-intervention | 0.000 (-0.1 – 0.1) | 0.996 |
| EEG – proportion of REM sleep | Post-intervention | -1.3 (-4.1 – 1.6) | 0.374 |
| EEG – proportion of deep sleep | Post-intervention | 4.9 (0.8 – 8.9) | **0.018*** |
| Subjective sleep quality (PSQI) | Post-intervention | -0.8 (-1.6 – 0.1) | 0.082 |
|  | Follow-up | 0.2 (-0.1 – 0.5) | 0.283 |
| Fatigue (FACIT-F, fatigue subscale) | Post-intervention | 2.9 (0.4 – 5.4) | **0.023*** |
|  | Follow-up | -0.8 (-1.6 – 0.07) | 0.071 |
| *IBD-related measures* | | | |
| Faecal calprotectin | Follow-up | -0.05 (-0.08 – -0.009) | **0.016*** |
| Haemoglobin | Follow-up | -0.03 (-0.07 – 0.007) | 0.109 |
| C-reactive protein | Follow-up | -0.007 (-0.03 – 0.02) | 0.551 |
| Albumin | Follow-up | 0.3 (-0.3 – 0.8) | 0.387 |
| Clinical index for disease activity in Crohn’s disease (HBI) | Post-intervention | -1.3 (-2.6 – 0.05) | 0.059 |
|  | Follow-up | 0.3 (-0.1 – 0.7) | 0.174 |
| Clinical index for disease activity in ulcerative colitis (SCCAI) | Post-intervention | 0.2 (-0.5 – 0.9) | 0.512 |
|  | Follow-up | -0.005 (-0.3 – 0.2) | 0.965 |
| Disease control (IBD control) | Post-intervention | -0.3 (-1.4 – 0.7) | 0.537 |
|  | Follow-up | 0.2 (-0.2 – 0.6) | 0.358 |
| IBD-related quality of life (SIBDQ) | Post-intervention | 1.6 (-0.4 – 3.6) | 0.120 |
|  | Follow-up | 0.4 (-0.4 – 1.1) | 0.342 |

*Abbreviations:* FACIT-F, Functional Assessment of Chronic Illness Therapy – Fatigue; FFMQ-SF, Five Facet Mindfulness Questionnaire-Short Form; HADS, Hospital Anxiety and Depression Scale; HBI, Harvey-Bradshaw Index; IBD, Inflammatory Bowel Diseases; MBCT, Mindfulness-Based Cognitive Therapy; MHC-SF, Mental Health Continuum-Short Form; PSQI, Pittsburgh Sleep Quality Inventory; PTQ, Perseverative Thinking Questionnaire; REM, rapid eye movement; SIBDQ, Short Inflammatory Bowel Disease Questionnaire; SCCAI, Simple Clinical Colitis Activity Index; SCS-SF, Self-Compassion Scale-Short Form; TAU, Treatment As Usual.

^a^TAU alone is reference category.

^b^Bold text plus an asterisk denotes statistical significance (p<0.05).

**Supplementary table S3: moderation analyses on pre-post changes in psychological distress (HADS) and well-being (MHC-SF)**

| Moderator | | Psychological distress (HADS) | | Well-being (MHC-SF) | |
| --- | --- | --- | --- | --- | --- |
|  | Reference category  (if applicable) | Unstandardised coefficient B^a^ (95% confidence interval) | *P value*^b^ | Unstandardised coefficient B^a^ (95% confidence interval) | *P value*^b^ |
| Age |  | 0.08 (-0.06 – 0.2) | 0.249 | 0.0005 (-0.02 – 0.02) | 0.954 |
| Sex | Women | 0.7 (-3.3 – 4.8) | 0.719 | -0.3 (-0.7 – 0.2) | 0.249 |
| Level of education | High education | Low: 4.9 (-1.9 – 11.7)  Medium: -0.7 (-4.6 – 3.4) | 0.158  0.741 | Low: 0.2 (-0.6 – 1.0)  Medium: 0.3 (-0.1 – 0.8) | 0.671  0.187 |
| IBD type | Ulcerative colitis | 0.1 (-3.8 – 4.0) | 0.948 | 0.1 (-0.3 – 0.6) | 0.554 |
| Disease duration |  | -0.06 (-0.2 – 0.1) | 0.496 | 0.03 (0.008 – 0.047) | **0.005*** |
| Medication use | No use | -0.1 (-4.5 – 4.2) | 0.954 | 0.1 (-0.2 – 0.5) | 0.849 |
| Repetitive negative thinking (PTQ T0) |  | -0.1 (-0.2 – 0.06) | 0.205 | 0.01 (-0.007 – 0.03) | 0.225 |
| Mindfulness skills (FFMQ T0) |  | 0.06 (-0.1 – 0.3) | 0.418 | -0.01 (-0.04 – 0.009) | 0.246 |
| Self-compassion (SCS T0) |  | -0.04 (-0.2 – 0.1) | 0.620 | -0.003 (-0.02 – 0.01) | 0.723 |
| Psychological distress (HADS T0) |  | -0.3 (-0.6 – -0.06) | **0.016*** | 0.03 (-0.002 – 0.06) | 0.063 |
| Well-being (MHC-SF T0) |  | 0.6 (-1.5 – 2.7) | 0.554 | -0.2 (-0.4 – 0.05) | 0.128 |

*Abbreviations*: FFMQ-SF, Five Facet Mindfulness Questionnaire-Short Form; HADS, Hospital Anxiety and Depression Scale; IBD, Inflammatory Bowel Diseases; -SF, Mental Health Continuum-Short Form; PTQ, Perseverative Thinking Questionnaire; SCS-SF, Self-Compassion Scale-Short Form; TAU, Treatment As Usual; T0, at baseline

^a^TAU alone is refence category.

^b^Bold text plus an asterisk denotes statistical significance (p<0.05).

**Supplementary table S4: mediation analyses on the effectiveness of MBCT on psychological distress**

A: univariate mediation analyses

| Mediator | Univariate mediation analyses^a^  (n=140) | Standardised coefficient β^b^ (95% CI) |
| --- | --- | --- |
| Mindfulness skills (FFMQ-SF)  (n=140) | **a: Group 🡪 FFMQ-SF** | **0.5 (0.2 – 0.8)*** |
|  | **b: FFMQ-SF 🡪 HADS** | **-0.3 (-0.5 – -0.2)*** |
|  | **c’: Group 🡪 HADS (corrected for FFMQ-SF)** | **-0.5 (-0.8 – -0.2) *** |
|  | **a*b: Group 🡪 FFMQ-SF 🡪 HADS** | **-0.2 (-0.3 – -0.05)*** |
| Self-compassion (SCS-SF)  (n=139) | **a: Group 🡪 SCS-SF** | **0.4 (0.05 – 0.7)*** |
|  | **b: SCS-SF 🡪 HADS** | **-0.2 (-0.4 – -0.06)*** |
|  | **c’: Group 🡪 HADS (corrected for SCS-SF)** | **-0.6 (-0.9 – -0.2)*** |
|  | **a*b: Group 🡪 SCS-SF 🡪 HADS** | **-0.08 (-0.2 – -0.005)*** |
| Repetitive negative thinking (PTQ)  (n=140) | a: Group 🡪 PTQ | -0.3 (-0.6 – 0.07) |
|  | **b: PTQ 🡪 HADS** | **0.5 (0.3 – 0.6)*** |
|  | **c’: Group 🡪 HADS (corrected for PTQ)** | **-0.5 (-0.8 – -0.2)*** |
|  | a*b: Group 🡪 PTQ 🡪 HADS | -0.1 (-0.3 – 0.03) |
| Sleep quality (PSQI)  (n=140) | a: Group 🡪 PSQI | -0.3 (-0.6 – 0.05) |
|  | **b: PSQI 🡪 HADS** | **0.3 (0.1 – 0.4)*** |
|  | **c’: Group 🡪 HADS (corrected for PSQI)** | **-0.6 (-0.9 – -0.3)*** |
|  | a*b: Group 🡪 PSQI 🡪 HADS | -0.07 (-0.2 – 0.01) |

*Abbreviations:* FFMQ-SF, Five Facet Mindfulness Questionnaire-Short Form; HADS, Hospital Anxiety and Depression Scale; PSQI, Pittsburgh Sleep Quality Inventory; PTQ, Perseverative Thinking Questionnaire; SCS-SF, Self-Compassion Scale-Short Form; TAU, Treatment As Usual.

^a^TAU alone is reference category.

^b^Bold text plus an asterisk denotes statistical significance (p<0.05).

B: multivariate mediation analysis including mindfulness skills and self-compassion

| Multivariate mediation analysis^†^ (n=139) | Standardised coefficient β (95% CI) |
| --- | --- |
| a1: Group 🡪 FFMQ-SF | **0.5 (0.1 – 0.8)*** |
| a2: Group 🡪 SCS-SF | **0.4 (0.05 – 0.7)*** |
| b1: FFMQ-SF 🡪 HADS | **-0.3 (-0.5 – -0.1)*** |
| b2: SCS-SF🡪 HADS | -0.07 (-0.2 – 0.1) |
| c’: Group 🡪 HADS (corrected for FFMQ-SF and SCS-SF) | **-0.5 (-0.8 – -0.2)*** |
| a*b: Group 🡪 FFMQ and SCS-SF 🡪 HADS | **FFMQ-SF: -0.1 (-0.3 – -0.03)***  SCS-SF: -0.03 (-0.1 – 0.03) |

*Abbreviations:* FFMQ-SF, Five Facet Mindfulness Questionnaire-Short Form; HADS, Hospital Anxiety and Depression Scale; SCS-SF, Self-Compassion Scale-Short Form; TAU, Treatment As Usual.

^a^TAU alone is reference category.

^b^Bold text plus an asterisk denotes statistical significance (p<0.05).
